# Survey of co-infection by *Salmonella* and oxyurids in tortoises

**DOI:** 10.1186/1746-6148-8-69

**Published:** 2012-05-28

**Authors:** Ludovico Dipineto, Michele Capasso, Maria Paola Maurelli, Tamara Pasqualina Russo, Paola Pepe, Giovanni Capone, Alessandro Fioretti, Giuseppe Cringoli, Laura Rinaldi

**Affiliations:** 1 Department of Pathology and Animal Health, Faculty of Veterinary Medicine, University of Naples Federico II, via della Veterinaria 1, 80137, Napoli, Italy

## Abstract

**Background:**

*Salmonella* spp. and oxyurids are among the most prevalent bacterial and parasitic agents in reptiles. These organisms are routinely isolated in healthy tortoises, although heavy infections may cause significant pathology. Tortoises are considered a common source of reptile-associated salmonellosis, an important zoonosis reported worldwide. A survey of the prevalence of *Salmonella* spp. and oxyurids in 53 tortoises was conducted in southern Italy and a possible correlation between the two pathogens was therefore investigated.

**Results:**

*Salmonella* spp. and oxyurids were detected with a prevalence of 49.1 and 81.1%, respectively. A significant positive correlation between *Salmonella* spp. and oxyurids was demonstrated. However, confounding factors related to husbandry could have been involved in determining this correlation.

**Conclusions:**

Our results suggest that caution should be exercised in translocation, husbandry, and human contact with tortoises and other exotic pets. Further studies on the epidemiology, molecular characterization and pathogenesis of *Salmonella* and oxyurids are needed to assess the actual impact of these organisms, as single or associated infections, on tortoises and on other exotic pets.

## Background

It is estimated that about 640 000 live reptiles are traded annually worldwide [1]. Although some species of tortoises are listed on Appendix II of the Convention on International Trade in Endangered Species (CITES) and thus subjected to strict trade regulations [2], there is a high demand for these reptiles to be kept as pets. Since the enactment of regulations implementing the CITES convention in the European Union (EU), several applications have been submitted for the CITES registration of privately owned tortoises in Italy; however, there are no official figures on the number of tortoises raised as pets in Italy [3]. The illegal introduction of reptiles raises public health concerns because these animals can be infected by various pathogens (virus, bacteria, protozoa, helminthes and arthropoda) and some of them are zoonotic [4].

Among bacteria, *Salmonella* spp. [5,6] is frequently reported in tortoises [6-8]. It is considered to be part of the intestinal flora and does not cause significant clinical sign, except in stressed or immunocompromised animals, where it may cause salmonellosis, an important reptilian zoonosis [8,9].

Several species of oxyurids commonly inhabit the colon of tortoises but are rarely considered pathogenic or zoonotic [10,11]. Heavy infections might be one cause of anorexia in tortoises coming out of hibernation [12]. Studies conducted so far in Italy showed oxyurids as the most prevalent nematode parasites in these animals [13-15].

We investigated the prevalence of *Salmonella* spp. and oxyurids in tortoises farmed in southern Italy. A secondary objective was to assess a possible correlation between the two pathogens investigated.

## Methods

### Ethics statement

Tortoises handling procedures were performed with the license of the farmers. The animals used in the present study were sampled following approval by the animal ethics and welfare committee of the University of Naples Federico II (in Italian, Comitato Etico-scientifico per la Sperimentazione Animale dell’ Università di Napoli Federico II; protocol number 0021099).

### Animals and sampling

Between May and August 2011, faecal samples were collected from 53 clinically healthy adult tortoises belonging to five species as indicated in Table 1. In particular, the species of tortoises examined were separated by species and were represented by: *Testudo hermanni* (22 *T. hermanni* ssp. *hermanni* and 8 *T. hermanni* ssp. *boettgeri)*, *Testudo marginata* (12 *T. marginata* ssp. *marginata*, and 1 *T. marginata* ssp. *weissingeri*)*, Testudo graeca* (7), *Testudo horsfieldii* (2), and *Geochelone carbonaria* (1).

**Table 1:** **Prevalence of *Salmonella *and oxyurids in the different species of tortoises farmed in southern Italy**

**Tortoise species**	**No. examined**	***Salmonella *spp.**			**Oxyurids**				**Correlation**
		**no. positives**	**%**	**95% CI**	**no. positives**	**%**	**95% CI**	**EPG**^**a**^	*Rho* =0.499 *P* = 0.000
*T. hermanni*	30	16	53.3	34.6 - 71.2	25	83.3	64.5 – 93.7	2462	
*T. marginata*	13	7	53.8	26.1 - 79.6	10	76.9	46.0 – 93.8	4735	
*T. graeca*	7	3	42.9	11.8 - 79.8	6	85.7	42.0 – 99.2	3756	
*T. horsfieldii*	2	0	-	-	2	100.0	19.8 – 95.1	664	
*G. carbonaria*	1	0	-	-	0	-	-	0	
**Total**	53	26	49.1	35.2 - 63.0	43	81.1	67.6 – 90.1	2323	

^a^ EPG = eggs per gram of faeces (calculated as mean for the positive values).

45 female and 7 male tortoises were examined and were selected from six private farms located in urban areas in the Campania region of southern Italy. Tortoises were housed in terrarium systems.

In order to perform bacteriological and parasitological analyses, each tortoise was lying carefully in a tank and one cloacal swab sample and one faecal sample for each reptile were collected. Cloacal swab samples were inoculated in Buffered Peptone Water (Oxoid, Milan, Italy), whereas faecal samples were preserved in formalin 5% and then stored at +4°C until further analyses.

### Bacteriological analysis

Cloacal swab samples were incubated at 37°C for 18 h. After incubation, samples were inoculated into Rappaport–Vassiliadis Broth (Oxoid) and incubated at 42°C for 18 h. Cultures obtained were plated onto Xylose-Lysine-Desoxycholate Agar (Oxoid), incubated at 37°C and examined after 24 h. Suspected *Salmonella* colonies were inoculated onto a second selective media, Brilliant Green Agar (Oxoid) and incubated at 37°C for 24 h. All isolates were biochemically identified by using the API20-E system (bioMérieux, Milan, Italy). All strains were stored frozen at –80°C in 20% glycerol until serotyping was performed. *Salmonella* isolates were serotyped according to the Kauffman-White scheme. Analyses were carried out in collaboration with the National Reference Laboratory for *Salmonella* (IZSVe, Legnaro, Italy).

### Parasitological analysis

Parasitological analyses were performed using the FLOTAC Pellet Technique [16]. This technique is performed for samples with an unknown weight of faecal material. In these circumstances, the weight of the faecal material to be analyzed can be inferred by weighing the sediment in the tube (pellet) after filtration and centrifugation of the faecal sample [17]. Formalin 5% was added to each faecal sample to reach a final volume of 20 ml; each sample was homogenized and filtered. Two 15 ml conical tubes were filled with the filtered suspension up to 6 ml and were centrifuged for 3 min at 1,500 rpm. After centrifugation the supernatant was discarded and the two pellets (sediments) were weighed. Two different flotation solutions were used to re-suspend the pellets: FS2 (Sodium Chloride Solution) (1200 s.g.) and FS7 (Zinc Sulphate Solution) (1350 s.g.). After homogenization, each of the two suspensions was poured into the two flotation chambers of the FLOTAC apparatus. The FLOTAC was closed and centrifuged for 5 min at 1,000 rpm; after centrifugation, the top parts of the flotation chambers were translated and each chamber was read under the microscope.

Parasitic elements (eggs, larvae, oocysts and cysts of oxyurids) were counted, photographed and measured using a light microscope at 10X and 40X magnifications (Leica DFC 490) and identified in accordance with Schneller and Pantchev [18].

#### Statistical analysis

For each tortoise, oxyurid eggs per gram (EPG) of faeces was calculated using the following formula: EPG = (N x 1.2)/wp where N is the number of eggs counted and wp is the weight of the pellet. Therefore, the mean EPG (for positive individuals) was calculated per each tortoise species. The relationship between *Salmonella* positivity and oxyurid EPG was evaluated by utilizing the Spearman’s *Rho* correlation performed using SPSS software (Version 13).

## Results

The results of the present study showed the presence of *Salmonella* spp. and oxyurids in all farms examined (6/6 = 100%) even though differences in prevalence values and EPG were recorded for each farm. *Salmonella* spp. was isolated from 26 out of 53 tortoises examined (49.1%; 95% confidence interval [CI] = 35.2 - 63.0%). Of the 26 *Salmonella* isolates, 23.1% (6/26) were serotyped as *S.* Richmond; 19.2% (5/26) as *S.* Abony; 15.4% (4/26) as *S.* Hermannswerder; 15.4% (4/26) as *S.* Lindern; 11.5% (3/26) as *S. enterica* subsp. *salamae* 6,7:z29:-:; 11.5% (3/26) as *S. enterica* subsp. *enterica* 9:a:1,5; 3.8% as *S.* Kottbus (1/26).

With respect to parasitological results, eggs of oxyurids were recorded in 43 out of the 53 tortoises examined (81.1%; 95% CI = 67.6 – 90.1%). Among the positive individuals, oxyurid EPG values ranged from 5 to 31,187. Table 1 indicates the mean oxyurid EPG for each tortoise species. Out of the 53 tortoises examined 23 (43.4%) were affected with both *Salmonella* spp. and oxyurids. The statistical analysis showed a significant positive correlation between oxyurid EPG and *Salmonella* (Spearman’s Rho = 0.499; *P* = 0.000).

The only *Geochelone carbonaria* tested was consistently negative both for *Salmonella* spp. and oxyurids. Other parasitic elements were also found in all the examined tortoises using the FLOTAC technique, indicating infection by ascarids, *Entamoeba* spp., *Nyctotherus* and *Balantidium coli*.

Results are summarized in Table 1.

## Discussion

The findings of the present study showed a prevalence of 49.1% for *Salmonella* spp. and 81.1% for oxyurids in tortoises farmed in southern Italy. Previous studies on the prevalence of *Salmonella* in tortoises conducted worldwide show heterogeneous results. Recovery rates of *Salmonella* in the present study are similar to the data reported by Savage and Baker [19] and Percipalle et al. [3] who reported a prevalence of 38.0% and 34.1%, respectively. Nevertheless, a recent survey conducted by Nowakiewicz et al. [20] showed levels of infection lower than those reported in the present study with a prevalence of 18.7%, whereas a study conducted by Pasmans et al. [7] showed levels of infection higher than those reported in our present study with a prevalence of 79.0%. The reasons for this recovery rate variation are not clear. The methods of isolation, species of tortoise, and geographical circumstances could have been involved in explaining these variable results.

The most prevalent *Salmonella* serotypes isolated in the present study were *S*. Richmond, *S*. Abony, and *S*. Hermannswerder which have been previously isolated in both captive and free-living tortoises [3,7,21]. It is noteworthy that some of the serotypes isolated during the present study have been associated with outbreaks of human salmonellosis worldwide. *S*. Kottbus have been associated with an outbreak in infants in Spain linked to contamination of commercial bottled water [22]. Another study conducted by Ryder et al. [23] suggests that *S*. Kottbus may colonize the human mammary gland and thus be transmitted to infants by breastfeeding. *S*. Richmond has been isolated during an outbreak of salmonellosis in a military detachment in Spain [24] and during an outbreak of dysentery in children in Malaysia [25].

It is not possible to speculate regarding the source of *Salmonella* infections in the present study because they may have been initiated before or after introduction of tortoises in the farms. The higher prevalence of certain serotypes compared to others could be a result of the potential cross-infection between animals which occurred during housing in the farms.

With respect to the parasitological analysis our results showed a prevalence of 81.1% for oxyurids; higher values compared with those recently reported in a study conducted by Papini et al. [15] who recorded a prevalence of 29.2% for these pinworms in captive tortoises farmed in central Italy. We can explain this difference in the prevalence rates by the different diagnostic tools used in the two studies. Papini et al. [15] used a routine faecal flotation method to detect helminthes. In contrast, we used the highly sensitive and multivalent FLOTAC technique which was also showed as the best copromicroscopic method for assessing pinworm prevalence as reported by Rinaldi et al. [26] in rabbits.

Interestingly, a significant positive correlation between *Salmonella* spp. and oxyurid EPG was observed in our study. Tortoises with high oxyurid EPG values were also positive to *Salmonella* spp. It is well demonstrated that parasites may be affected, directly or indirectly, by cytokines and other immune effector molecules and parasites may themselves produce factors that affect the cells of the immune system [27]. The consequent immunomodulation may enhance host susceptibility to other infectious pathogens [27] such as *Salmonella* spp. Furthermore, the interactions between parasites and other infectious agents have been described in various surveys in literature. Cringoli et al. [28] reported the co-presence of antibodies to *Neospora caninum* and *Leishmania infantum* in dogs suggesting that one protozoan may enhance the susceptibility to other one. Rinaldi et al. [29] showed *N. caninum* and Bovine herpesvirus 1 (BHV-1) coinfection in cattle, demonstrating the role of *N. caninum* as a primary pathogen and its presence as a risk factor for BHV-1 infection in this animal species. These studies, in line with the present study, suggest that in mixed infections the burden of one or both the infectious agents may be increased, one or both may be suppressed or one may be increased and the other suppressed. However, the correlation found between *Salmonella* and oxyurids in the present study does not necessarily reflect a causal relationship between the two organisms. Indeed confounding factors related to husbandry could have been involved in determining this correlation. It is very likely that this correlation might be also enhanced by the habitat (sanitation, temperature, diet) of the tortoises and further studies are needed to address this issue.

## Conclusions

In conclusion, the findings of the present study showed a significant positive correlation (Spearman’s Rho = 0.499; *P* = 0.000) between *Salmonella* and oxyurid infection in tortoises farmed in southern Italy. Further studies on the epidemiology, molecular characterization and pathogenesis of *Salmonella* and oxyurids, as well as on the role of husbandry in the correlation by the two pathogens, are needed to assess the actual impact of these organisms, as single or associated infections, on tortoises and other exotic pets. In addition, as direct transmission of pathogens (as *Salmonella*) to humans may occur through handling of an infected reptile and indirect transmission may occur through contact with feces or by handling objects contaminated by reptiles, results presented here suggest that those individuals who handle tortoises should emphasize sanitary precautions.

## Consent

Consent was obtained from the owner of the animal for publication of this study.

## Competing interests

The authors declare that they have no competing interests.

## Authors’ contributions

LD and LR conceived the study, designed the experiments, analyzed the experimental data and prepared the manuscript. AF and GC2 supervised the experiments and helped draft manuscript. MPM and PP performed the parasitological analysis. TPR performed the bacteriological analysis. MC and GC1 performed the sampling. All authors read and approved the final manuscript.
